# *Apolipoprotein E* Overexpression Is Associated With Tumor Progression and Poor Survival in Colorectal Cancer

**DOI:** 10.3389/fgene.2018.00650

**Published:** 2018-12-13

**Authors:** Zhixun Zhao, Shuangmei Zou, Xu Guan, Meng Wang, Zheng Jiang, Zheng Liu, Chunxiang Li, Huixin Lin, Xiuyun Liu, Runkun Yang, Yibo Gao, Xishan Wang

**Affiliations:** ^1^Department of Colorectal Surgery, The Second Affiliated Hospital of Harbin Medical University, Harbin, China; ^2^Department of Pathology, National Cancer Center/National Clinical Research Center for Cancer/Cancer Hospital, Chinese Academy of Medical Sciences and Peking Union Medical College, Beijing, China; ^3^Department of Colorectal Surgery, National Cancer Center/National Clinical Research Center for Cancer/Cancer Hospital, Chinese Academy of Medical Sciences and Peking Union Medical College, Beijing, China; ^4^Department of Thoracic Surgery, National Cancer Center/National Clinical Research Center for Cancer/Cancer Hospital, Chinese Academy of Medical Sciences and Peking Union Medical College, Beijing, China; ^5^Genesis (Beijing) Co., Ltd., Beijing, China

**Keywords:** colorectal cancer, *Apolipoprotein E* (*ApoE*), prognosis, stage II, simultaneous liver metastasis, biomarkers, chemotherapy

## Abstract

*Apolipoprotein E* (*ApoE*) plays a key role in tumorigenesis and progression, such as cell proliferation, angiogenesis and metastasis. *ApoE* overexpression was associated with aggressive biological behaviors and poor prognosis in a variety of tumor according to previous studies. This study aimed to assess the prognostic value and explore the potential relationship with tumor progression in colorectal cancer (CRC). We collected the expression profiling microarray data from the Gene Expression Omnibus (GEO), investigated the *ApoE* expression pattern between the primary CRC and liver metastasis of CRC, and then explored the gene with prognostic significance based on the TCGA database. *ApoE* high expression was associated with poor overall survival (OS, *p =* 0.015) and progression-free survival (PFS, *p =* 0.004) based on the public databases. Next, ApoE expression was evaluated in two CRC cohorts by immunohistochemistry, of whom 306 cases were stage II and 201 cases were metastatic liver CRC. In the cohort of the liver metastasis, the *ApoE* expression was increasing in normal mucosa tissue, primary colorectal cancer (PC), and colorectal liver metastases (CLM) in order. Meanwhile, the level of *ApoE* expression in stage II tumor sample which had no progression evidence in 5 years was lower than that in PC of synchronous liver metastases. The high *ApoE* expression in PC was an independent risk factor in both stage II (HR = 2.023, [95% CI 1.297–3.154], *p =* 0.002; HR = 1.883, [95% CI 1.295-2.737], *p =* 0.001; OS and PFS respectively) and simultaneous liver metastasis (HR = 1.559, [95% CI 1.096–2.216], *p =* 0.013; HR = 1.541, [95% CI 1.129–2.104], *p =* 0.006; OS and PFS respectively). However, the overexpression of *ApoE* could not predict the benefit from the chemotherapy in stage II. The study revealed that the relevance of the *ApoE* overexpression in CRC progression, conferring a poor prognosis in CRC patients especially for stage II and simultaneous liver metastasis. These finding may improve the prognostic stratification of patients for clinical strategy selection and promote CRC clinic outcomes.

## Introduction

Colorectal cancer (CRC) is one of the most common digest track malignant tumors, which are threatening the public health worldwide. According to the data published by Chinese National Cancer Center, in China, over 376.3 thousand CRC new cases and 191.0 thousand CRC-related deaths were estimated just in 2015 (Chen et al., 2016).The current treatment regimen option mainly depends on American joint committee on cancer TNM staging classification system which is based on the clinicopathologic characteristics. However, owing to the tumor heterogeneity, the patients with the same staging and similar treatment may gain different clinical outcomes. Moreover, chemotherapy as one of principal therapeutic means is recommended for stage III, IV and part of II CRC patients according to the CRC treatment guideline. Regarding stage II CRC, chemotherapy could improve survival outcome of patients, but absolute improvement in survival was less than 5% (Chen et al., 2016). Adverse events from adjuvant chemotherapy would have impacts on the quality of life of patients (Rosmarin et al., 2014). Therefore, there remains an urgent to identify valuable biomarkers aiming to improve the prognostic stratification of patients for clinical strategy selection.

*Apolipoprotein E* (*ApoE*) plays a multi-functional role in cholesterol transport and metabolism, which mediates the cellular uptake of lipoprotein particles by binding to receptors of low-density lipoprotein (LDL) receptor family and the receptor for chylomicron remnants (Gliemann, 1998). Previous research has suggested *ApoE* abnormal function is associated with Alzheimer’s disease, atherosclerosis and chronic heart disease (Wilson et al., 1996; Hofman et al., 1997; Greenow et al., 2005). Besides, the functions of *ApoE* have been identified in DNA synthesis, cell proliferation, angiogenesis and metastasis, so the aberration of these functions may lead to tumorigenesis and progression. *ApoE* overexpression has previously been reported in gastric, lung, prostate, thyroid, ovarian, endometrial cancer and glioblastoma (Nicoll et al., 2003; Venanzoni et al., 2003; Oue et al., 2004; Ito et al., 2006; Huvila et al., 2009; Su et al., 2011). A recent study has shown that *ApoE* was associated with tumor advanced grade and stage in gastric carcinomas and involved in invasion, metastasis and carcinogenesis (Oue et al., 2004). Another study found that increased expression of *ApoE* might represent a late event in the progression of endometrioid endometrial adenocarcinoma (Huvila et al., 2009). In lung adenocarcinoma, *ApoE* over-expression promotes cancer proliferation and migration and is related to chemo-resistance (Su et al., 2011). A recent study implicated the *ApoE* moderates the colon homeostasis and constitutes a risk factor for colon pathologies (El-Bahrawy et al., 2016). However, the prognostic value of *ApoE* expression for CRC remains unclear, and to the best of our knowledge, although some previous research has been implicated that APOE might influence CRC development through three potential path ways: cholesterol and bile metabolism, triglyceride and insulin regulation, and the prolonged inflammation (Slattery et al., 2005; Mrkonjic et al., 2009; Kato et al., 2010), there has not been a prior study of functional expression and prognostic significance for CRC.

In the present study, we analyzed Affymetrix gene microarray in the setting of liver metastatic CRC from the GEO, which aimed to study the expression pattern of *ApoE* between CRC primary and liver metastasis samples. Next, we evaluated the expression patterns of *ApoE* in CRC and assessed prognostic significance based on The Cancer Genomic Atlas (TCGA). Subsequently, we further studied the expression pattern in stage II and liver metastasis of CRC respectively, and made survival analysis in two cohorts, to explore the relationship of the expression features with the clinic and prognosis.

## Materials and Methods

### Patients and Tissue Samples

The specimens in this study were collected from the CRC patients who underwent the surgical resection from January 2006 through December 2012, which were all archived by Pathology Department of Cancer Institute and Hospital, Chinese Academy of Medical Sciences. All the sample diagnoses were confirmed according to the 7th edition of TNM staging system. The inclusive criteria of stage II were as follow: (A) AJCC pathology staging was stage II (T3-4N0M0); (B) no systemic or chemotherapy before the surgery; (C) the case can provide complete clinical information, such as age, gender, tumor location, histology, differentiation, TNM classification, adjuvant therapy regime, follow-up information and so on. At the same time, we utilized the primary tumor and corresponding metastatic liver specimen to establish another simultaneous liver metastatic CRC cohort (LMCRC). Totally, 306 cases of stage II CRC and 201 cases of liver metastatic CRC were collected based on the inclusive criteria. The Clinical Research Ethics Committee of Cancer Institute and Hospital, Chinese Academy of Medical Sciences approved this study. All the patients were followed up regularly until December 31st, 2017, every 3 months up to the 5th year.

### *ApoE* Expression Analyses in the GEO and TCGA Databases

To investigate the *ApoE* expression pattern between the primary CRC (PC) and liver metastasis of CRC (CLM), we collected the expression profiling microarray data from the Gene Expression Omnibus (GEO^1^) database under the accession number GSE41258 (Affymetrix Human Genome U133A Array), GSE62322 (Affymetrix Human Genome U133B Array) and GSE68468 (Affymetrix Human Genome U133A Array), respectively. Gene expression was first measured at the probe set level using the RMA (Robust Multi-array Average) methodology on perfect match probes, followed by quantile normalization. Probe set annotation for the U133 Array was downloaded from Affymetrix’s website. The probe set with the greatest average expression across all samples was chosen to represent each gene. Information about datasets was summarized in Supplementary Table S1. All the sample preparation and microarray were performed based on the standard protocols. The standardized *ApoE* expression was obtained by dividing into N (normal mucosa), PC and CLM in each of datasets after annotation.

Two hundred and seventy one cases of colon cancer and 89 cases of rectal cancer are provided by the TCGA project (Supplementary Table S8). According to the expression value of *ApoE*, the cohort was classified into high expression group and low expression group (cut-off = 50%) after merging the colon and rectal cancer cases. The Box Plots was generated to compare the *ApoE* expression level between the tumor and normal tissues of CRC, and to show the *ApoE* expression features in different pathological stages. A tool named GEPIA^2^ which is an interactive web server for analyzing the RNA sequencing expression data from the TCGA projects is used for batch TCGA data processing and visualization in this study (Tang et al., 2017).

### Tissue Microarray and Immunohistochemistry

The stage II cohort included the tumor and normal tissue of each patient, and LMCRC cohort consisted of the primary tumor, metastatic tumor, normal intestinal mucosa and normal liver tissue from each patient. The TMAs were built after verification by HE staining and the punched sample which measured 1.0 mm were taken from the center of the tumor. The different specimen derived from one patient were placed on the same TMA and every TMA has another copy to reduce systematic errors.

**FIGURE 1:**
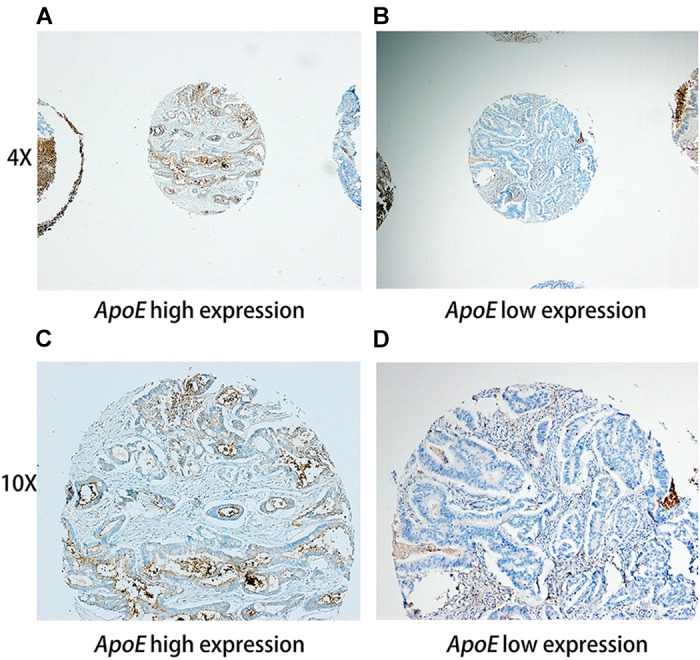
Representative immunohistochemistry staining pictures of ApoE expression in CRC tissues Tissue high expression (4X for **A**, 10X for **C**) and low expression (4X for **B**, 10X for **D**) for the ApoE protein are shown. Each of punched samples is 1.0 mm in the tissue microarrays.

Immunohistochemical staining was performed on the slides (5 μm thick) from the TMAs, using an ApoE (pan) (D7I9N) rabbit monoclonal antibody (#13366; 1:500; Cell Signaling Technology, United States) antibody to ApoE, as it was described previously (Holtzman et al., 2000). The SI score was calculated by multiplication of the staining intensity (0, negative; 1, weak; 2, moderate; 3, strong) and the percentage of positive stained cells (no staining, 0; 1–10%, 1; 11–50%, 2; 50–100%, 3). In this study, moderate/strong cytoplasm staining of (SI = 3–9) was defined as positive staining, while weak or negative staining (SI = 0–2) was defined as negative staining. Representative staining of ApoE in the specimens illustrated in Figure 1. Positive rate refers to the proportion of *ApoE* positive staining samples, namely positive rate = positive samples/(positive samples + negative samples).

### Statistical Analysis

The statistical significance of the difference was assessed using Student *t*-test, and the one-way ANOVA with Tukey post-test was conducted for multiple comparisons. Chi-square test or Fisher exact test was used to evaluate the difference in rates among different groups. All the statistical results were summarized in Supplementary Table S9. Survival curves were plotted according to the Kaplan–Meier method and the log-rank test was used to compare the overall survival (OS) and progression free survival (PFS) in the study cohort. Univariate and multivariate analysis for CRC prognosis were undertaken using Cox proportional hazards regression model. The calculations were performed with IBM SPSS Statistics 24.0 software program and R version 3.3.3. A value of *p <* 0.05 was considered as significant.

## Results

### *ApoE* Is Highly Expressed in Colorectal Liver Metastasis and Has Prognostic Significance in Colorectal Cancer Based on the Public Databases

**FIGURE 2:**
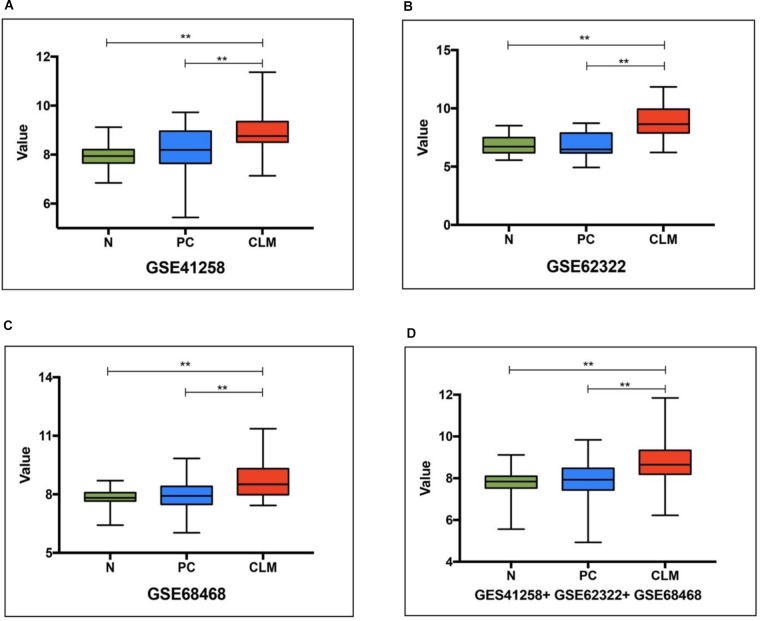
*ApoE* expression pattern in the normal intestinal mucosa, primary tumor and colorectal liver metastasis based on 3 datasets (**A**, GSE41258, **B**, GSE62322, **C**, GSE68468) from GEO and their pooling set **(D)**. N, normal intestinal mucosa; PC, primary colorectal cancer; CLM, colorectal liver metastasis; ^∗∗^Represents *p*-value < 0.01.

We first assessed the *ApoE* expression level in the normal intestinal mucosa, PC and CLM based on 3 datasets from GEO (Supplementary Table S1). In GSE41258 and GSE 62322, PC refers to the primary tumor from metastatic CRC, but to account for the limit of clinical data PC in GSE68468 included all the stages. As demonstrated in Figure 2, *ApoE* was significantly higher expressed in CLM compared with normal tissue and PC in all three datasets. However, there was no significant difference between the normal mucosa and PC.

**FIGURE 3:**
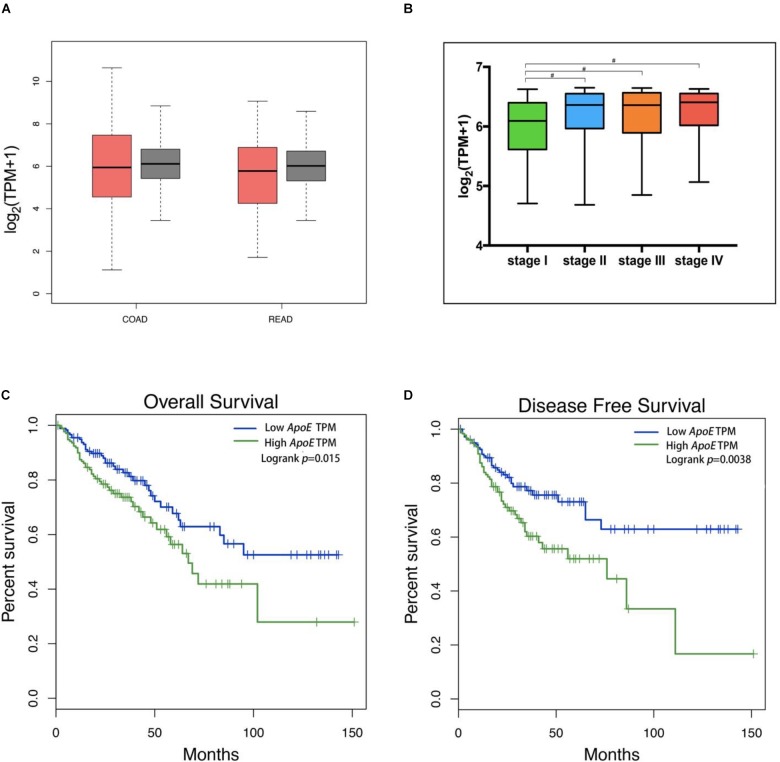
Expression of the ApoE protein in CRC and Kaplan–Meier Curves based on TCGA database. The tumor was represented by red color and the normal tissue was represented by gray color **(A)**. The ApoE expression box plots were generated based on CRC patient pathological major TNM staging **(B)**. The most extreme value from bottom to top in the box plot represents minimum value, the lower quartile, the median, the upper quartile and the maximum value. The method for differential gene expression analysis is one-way ANOVA, using the pathological stage as a variable for calculating differential expression. The ApoE high expression group was associated with decreased overall survival **(C)** and disease-free survival **(D)** in CRC according to the data from TCGA, which were calculated using a log-rank test. CRC, Colorectal cancer; TPM, transcript per million; ^#^Represents *p*-value < 0.05.

To further investigate the *ApoE* expression pattern and prognostic significance in CRC, we analyzed the expression of *ApoE* in TCGA database. The result revealed that there was no significant difference between the PC and normal tissues in both the colon cancer (COAD) and rectal cancer (READ) dataset, which was consistent with GEO data (Figure 3A). In the meanwhile, the *ApoE* expression demonstrated rising tendency in general as the pathology stage development and *ApoE* expression level in stage I was significantly lower than the other stages (Figure 3B). As illustrated in the Kaplan–Meier survival curves, overexpression *ApoE* proved to associated with poorer OS and the DFS in CRC patients (*p* = 0.015 for OS; *p =* 0.004 for DFS; Figures 3C,D).

### The *ApoE* Expression Features in the LMCRC and the Stage II CRC Cohort

According to the results analyzed from the public data, we primary identified the expression patterns and the potential prognostic value of *ApoE* in CRC. Therefore, we further investigated the expression patterns of *ApoE* in 201 cases of PC and CLM from simultaneous liver metastasis patients and corresponding adjacent normal mucosa and liver tissues utilizing immunohistochemistry staining. As shown in Table 1, ApoE protein expression was detected in 103/201 (51.2%) of the PC samples, 128/201 (63.7%) of the CLM samples and 43 cases (21.4%) of adjacent normal mucosa stained positively. Thus, at protein levels, the expression of ApoE was higher than normal mucosa (51.2% vs. 21.4%, *p <* 0.001) and ApoE was upregulated in the CLM tissues (63.7% vs. 51.2%, *p =* 0.012) comparing with PC.

**Table 1:** The ApoE expression pattern in different samples by IHC staining.

	Sample	ApoE Positive	ApoE Negative	Positive Rate %
Stage II	Tumor	105	201	34.3
	Normal tissue	120	186	39.2
	Progression	51	60	45.9
	Non-progression	54	141	27.7
	Recurrence of liver metastasis	16	14	53.3
	after surgery
LMCRC	Primary tumor	103	98	51.2
	Normal colorectal mucosa	43	158	21.4
	Liver metastasis	128	73	63.7
	Normal liver tissue	89	112	44.3


*Stage II, stage II colorectal cancer; LMCRC, liver metastatic colorectal cancer; Progression, tumor recurrence after surgery in 5 years; Non-Progression, no recurrence signs after surgery in 5 years.*

In the cohort of stage II, there was no significant difference between the tumor and normal tissue (34.3% vs. 39.2%, *p =* 0.209). According to the follow-up, 306 cases of the stage II patients were divided into the non-progression group (195 cases) and the progression group (111 cases), and 30 cases with liver metastasis after surgery included. Immunohistochemistry staining indicated that progression group had a higher ApoE expression positive rate than the non-progression group (45.9% vs. 27.7%, *p =* 0.001). Comparing the *ApoE* expression level of the primary tumor between stage II and simultaneous liver metastatic group, the latter turned out to be higher (34.3% vs. 51.2%, *p =* 0.001). We further analyzed the *ApoE* expression pattern in primary tumor between the stage II with liver metastasis after surgery and the simultaneous liver metastatic group, whereas it proved no significant difference (53.3% vs. 51.2%, *p =* 0.831).

### The Low *ApoE* Expression Is Associated With Improved Survival Outcome in Two Cohorts

Two cohorts of CRC patients were classified into low *ApoE* expression group (SI 0–4) and high *ApoE* expression group (SI 6–12) based on the immunohistochemistry staining of the primary tumor. The relationship between the *ApoE* expression and the clinicopathologic characteristics of stage II and LMCRC patients are summarized in Supplementary Tables S2, S3, respectively. *ApoE* highly expressed in the LMCRC cohort patients who underwent neoadjuvant therapy. Besides, the other clinicopathologic information such as age, gender, tumor location, gross pathology type, differentiation grade, T stage, MSI (Microsatellite instability) status, preoperative CEA level and preoperative CA19-9 level had no significant correlation with the *ApoE* expression in both two cohorts (Supplementary Tables S2, S3).

**FIGURE 4:**
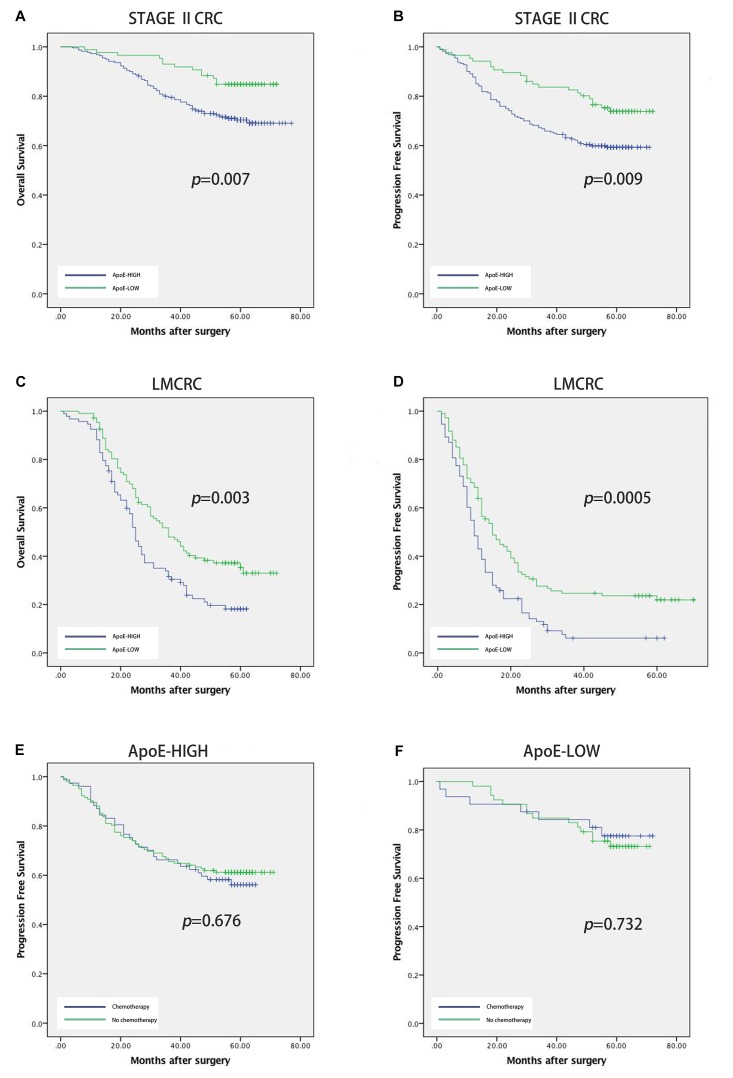
Prognostic power of *ApoE* in stage II CRC cohort and liver metastatic CRC cohort. Kaplan–Meier analyses of overall survival and progression free survival in patients with CRC based on the expression of *ApoE*. OS and PFS according to *ApoE* expression in stage II CRC **(A,B)** and liver metastatic CRC **(C,D)**. The relationship between *ApoE* expression and PFS benefit from adjuvant chemotherapy in patients with stage II CRC. Treatment with 5-FU based chemotherapy was not associated with a higher rate of PFS both in the *ApoE* high group **(E)** and the *ApoE* low group **(F)**.

To identify the prognostic significance of the *ApoE* expression in CRC, we further conducted the survival analysis in two cohorts respectively. In the cohort of stage II, the median follow-up was over 59 months, 78 died cases and 111 relapsed patients included. Kaplan–Meier curves revealed that the patients with low *ApoE* expression had a longer 5-year OS and PFS (*p* = 0.002 for OS and *p =* 0.001 for PFS; Figures 4A,B) in stage II cohort. Multivariate Cox regression analysis confirmed that high *ApoE* was independently associated with worse prognosis significance for OS (HR 2.023, [95% CI 1.297–3.154]) and PFS (HR 1.883, [95% CI 1.295–2.737]) (Tables 2, 3 and Supplementary Tables S4, S5). MSI status was independently associated with better OS (HR 0.328, [95% CI 0.120–0.897]) and neurological involvement was an independent prognostic factor for PFS in multivariate analysis (HR 2.115, [95% CI 1.133–3.949]).

**Table 2:** Cox analyses of potential prognostic factors for overall survival in the stage II CRC cohort.

Factor	Comparison	Univariate Analysis			Multivariate Analysis		
		HR	95%CI	*p-*value	HR	95%CI	*p*-value
MSI status	MSI vs. MSS	0.343	0.126–0.939	0.037	0.328	0.120–0.897	0.030
*ApoE* expression	HIGH vs. LOW	1.973	1.266–3.077	0.003	2.023	1.297–3.154	0.002


**Table 3:** Cox analyses of potential prognostic factors for progression-free survival in the stage II CRC cohort.

Factor	Comparison	Univariate Analysis			Multivariate Analysis		
		HR	95%CI	*p-*value	HR	95%CI	*p*-value
Neurological Involvement	Present vs. Absent	2.222	1.191–4.145	0.012	2.115	1.133–3.949	0.019
*ApoE* expression	HIGH vs. LOW	1.913	1.317–2.780	0.001	1.883	1.295–2.737	0.001


Kaplan–Meier analysis was also conducted in simultaneous liver metastatic patients. With 27-month median follow-up, 141 patients died and 168 patients relapsed. *ApoE*-low group had a significantly improved OS (*p* = 0.002 for OS and *p =* 0.008 for PFS; Figures 4C,D). The multivariate analysis demonstrated that *ApoE* expression in PC was an independent prognosticator for OS (HR 1.559, [95% CI 1.096–2.216]) and PFS (HR 1.541, [95% CI 1.129–2.104]) in patients with synchronous liver metastasis CRC (Tables 4, 5 and Supplementary Tables S6, S7). Besides, in the LMCRC cohort, N staging was an independent prognostic indicator in both OS (HR 0.488, [95% CI 0.302–0.789]) and PFS (HR 0.462, [95% CI 0.302–0.706]).

**Table 4:** Cox analyses of potential prognostic factors for overall survival in the simultaneous liver metastatic CRC cohort.

Factor	Comparison	Univariate Analysis			Multivariate Analysis		
		HR	95%CI	*p-*value	HR	95%CI	*p*-value
T Stage	T1-3 vs. T4	0.696	0.499–0.970	0.032	0.786	0.556–1.109	0.170
N Stage	N0 vs. N+	0.502	0.312–0.808	0.005	0.488	0.302–0.789	0.003
Chemotherapy	Yes vs. No	0.641	0.429–0.956	0.029	0.766	0.512–1.146	0.195
*ApoE* expression	HIGH vs. LOW	1.629	1.163–2.281	0.005	1.559	1.096–2.216	0.013


**Table 5:** Cox analyses of potential prognostic factors for progression-free survival in the simultaneous liver metastatic CRC cohort.

Factor	Comparison	Univariate Analysis			Multivariate Analysis		
		HR	95%CI	*p-*value	HR	95%CI	*p*-value
N stage	N0 vs. N+	0.483	0.317–0.737	0.001	0.462	0.302–0.706	<0.001
MSI	MSI vs. MSS	0.497	0.262–0.942	0.032	0.555	0.291–1.057	0.073
*ApoE* expression	HIGH vs. LOW	1.496	1.100–2.033	0.010	1.541	1.129–2.104	0.006


### The Expression of *ApoE* Could Not Predict the Benefit From the Adjuvant Chemotherapy for Stage II CRC

Next, we investigated the potential role of *ApoE* as a predictor of adjuvant chemotherapy for stage II. In the stage II cohort, 131 patients received the 5-FU-based adjuvant chemotherapy, 63 lower rectal cancer patients underwent radiotherapy (50Gy) and 112 patients underwent surgery alone. *ApoE* expression was shown to have a negative impact on survival both the patients who underwent surgery alone (25.5% vs. 43.9%, *p =* 0.049) and those who received the 5-FU-based chemotherapy (53.3%vs. 73.3%, *p =* 0.019) (Figures 4E,F). We explored the association between *ApoE* expression and PFS among the patients who either received or did not receive the chemotherapy. However, there was no significant interaction between the chemotherapy and high *ApoE* expression of CRC. Further analysis showed the benefit observed in high *ApoE* expression group was superior to that in low expression group.

## Discussion

In the present study, we studied the *ApoE* expression profiling and relevant prognostic value of *ApoE* in CRC, especially for stage II and liver metastasis. We compared the expression level of *ApoE* in primary lesion, liver metastases and corresponding normal mucosa according to three GEO datasets and two our center cohorts. We found that *ApoE* was significantly higher expressed in CLM compared with normal tissue and PC. Here, we proposed an assumption that the different expressing genes between the CRC primary and liver metastatic tumors may play roles in the metastasis or progression and these genes would have the potential prognostic value. We found that *ApoE* expression level proved rising tendency in stage II tumor, primary tumor and liver metastasis of CLM in order, and high *ApoE* expression was associated with shorter PFS in stage II cohort. Thus we conducted survival analysis based on TCGA data and validated the result in our two cohorts. When we made survival analysis based on the TCGA data, it was demonstrated that the expression of *ApoE* was significantly associated with OS and PFS of CRC. Next, the survival analysis was performed in the two cohorts to validate the prognostic significance in stage II and metastatic CRC. In two cohorts, the higher expression level of *ApoE* has been shown to be independently associated with a reduced prognosis. Besides, neurological involvement was also independently related to the PFS of stage II. Concerning liver metastasis of CRC, we found that N staging was one of the independent risk factors both in OS and PFS. The patients should be stratified based on the independent prognostic factor to accept suitable treatment regime.

Previous studies have demonstrated the overexpression of *ApoE* was associated with a series of malignant behaviors and it was regarded as a prognostic marker in a variety of cancers according to previous studies (Nicoll et al., 2003; Oue et al., 2004; Ito et al., 2006). Related studies have revealed that *ApoE* activity on cancer cells is dual according to different tissues and *ApoE* affects several signaling cascades, including by increasing disabled phosphorylation and by activation of the *ERK1/2* pathway (Hoe et al., 2005; Zheng et al., 2018). At the same time, *ApoE* could activate *PI3K/AKT/mTOR* signaling pathway, which has been confirmed as a critical regulator during tumor progression, including cell–cell adhesion, proliferation, and migration (Thorpe et al., 2015). Meanwhile, the aberration of *ApoE* expression might also lead to the development of CRC. Niemi et al. (2002) found HT29 cell line with overexpressed *ApoE* would enhance the cell polarity which was one potential step of tumor metastasis. Mrkonjic et al. (2009) reported that *ApoE*-expressing cell would induce proliferative signals and inhibit apoptosis in CRC. These findings suggested that *ApoE* might be a potential predictive marker during the development of CRC. Intriguingly, in our cohorts *ApoE* was highly expressed in liver metastasis than primary tumor, however, there was no significant difference in primary lesion between the stage II and stage IV according to the TCGA data. Because stage IV samples in TCGA databases were not the only liver metastasis but also included the other types of metastatic CRC. Consequently, we suspect that *ApoE* may be one of the potential liver metastasis-specific biomarkers in CRC, but this assumption remains to be further verified by the larger sample scale.

However, it turned out that *ApoE* had a prognostic rather than predictive value, which did not seem to be associated with the resistance to chemotherapy in stage II. Even so, we demonstrated that stage II CRC with overexpressed *ApoE* was more prone to recurrence or metastasis and worse prognosis. The results remind us stage II patients should increase the postoperative follow-up frequency properly according to the *ApoE* expression level. The study indicated the association between the high *ApoE* expression and MSS (Microsatellite Stability) status in stage II. Previous studies have shown that the prognosis of with MSI is better than those with MSS for the stage II patients (Salazar et al., 2011) and the result was also confirmed in our study. It may include that some interactions between the *ApoE* expression level and DNA mismatch-repair (MMR) functional status, which needs to be further explored and identified. The results also showed that the neoadjuvant therapy for liver metastasis significantly increased the *ApoE* expression level. We hypothesize that high-dozen chemotherapy might lead to metabolic abnormality of lipid through the body and therefore a high level of *ApoE* was detected in CRC. The alternative of *ApoE* after neoadjuvant chemotherapy cannot reflect the real expression level in the tumor. Consequently, if we intend to take the *ApoE* as a prognostic marker, the effects from preoperative chemotherapy should be taken into account in advance. Besides, we think *ApoE* could be considered as a valuable molecular marker for prediction of CRC prognosis and it might be a potential new therapeutic target of the CRC.

One of the limitations of this study is that we did not detect the presence of three common isoforms, including ApoE2, E3 and E4 which are from amino acid substitutions (Raffai et al., 2001). Different ApoE isoforms by binding to the LDL receptor could lead to various of biological behaviors for tumor, so the related research stratified by *ApoE* phenotypes is required. On the other hand, although the result analyzed by TCGA indicated that the *ApoE* expression level increases with the development of CRC and *ApoE* have potential prognostic value in CRC, our validation cohorts only consist of stage II and liver metastatic CRC patients. Especially the TCGA data showed that *ApoE* expression level in stage I was significantly lower than the other stages. Whether the *ApoE* could be regarded as a potential biomarker for diagnosis or *ApoE* plays some critical roles in the development from stage I to the higher stage, it should be further verified. Therefore, next, we need to complete the CRC cohort establishment of stage I, stage III and even precancerous, in order to further vindicate current results.

## Conclusion

In this study, we found that the *ApoE* expression was higher in the primary tumor of liver metastasis as compared with the stage II. High level of *ApoE* was an independent prognostic indicator for OS and PFS in stage II and simultaneous liver metastatic CRC.

## Ethics Statement

This study was carried out in accordance with the recommendations of 7th edition of TNM staging system, the Clinical Research Ethics Committee of Cancer Institute and Hospital, Chinese Academy of Medical Sciences with written informed consent from all subjects. All subjects gave written informed consent in accordance with the Declaration of Helsinki. The protocol was approved by the Clinical Research Ethics Committee of Cancer Institute and Hospital, Chinese Academy of Medical Sciences.

## Author Contributions

ZZ and YG designed the study. ZZ, SZ, XG, ZL, and YG collected the data. ZZ, SZ, XG, and YG analyzed the data. SZ, XG, and YG interpreted the data. MW, ZJ, ZL, HL, and XL sourced the literature. ZZ, SZ, MW, and ZJ wrote the draft. CL and RY edited the manuscript. XW acquired the funding and supervised the whole study.

## Conflict of Interest Statement

The authors declare that the research was conducted in the absence of any commercial or financial relationships that could be construed as a potential conflict of interest.

## Footnotes

1 http://www.ncbi.nlm.nih.gov/geo/

2 http://gepia.cancer-pku.cn/index.html
