# Supplementary material for: Apolipoprotein E Overexpression Is Associated With Tumor Progression and Poor Survival in Colorectal Cancer
Source: Front Genet. 2018 Dec 13;9:650. doi: 10.3389/fgene.2018.00650 (PMC6315167; doi:10.3389/fgene.2018.00650)
Supplement: Supplementary file 1 [file Table_1.DOCX]

| **Table S1**  **Relative information of the datasets from the GEO database** | | | | |
| --- | --- | --- | --- | --- |
| **GSE** | **Platform** | **Normal Mucosa** | **Primary Tumor** | **Colorectal Liver Metastasis** |
| **GSE41258** | Affymetrix Human Genome U133A Array | 54 | 58 | 47 |
| **GSE62322** | Affymetrix Human Genome U133B Array | 18 | 20 | 19 |
| **GSE68468** | Affymetrix Human Genome U133A Array | 55 | 195 | 47 |
| Primary Tumor in GSE41258 and GSE 62322 represented only the primary lesion from metastatic colorectal cancer, whereas GSE68468 included all the stages of colorectal cancer. | | | | |
