# Supplementary material for: Apolipoprotein E Overexpression Is Associated With Tumor Progression and Poor Survival in Colorectal Cancer
Source: Front Genet. 2018 Dec 13;9:650. doi: 10.3389/fgene.2018.00650 (PMC6315167; doi:10.3389/fgene.2018.00650)
Supplement: Supplementary file 2 [file Table_2.DOCX]

| **Table S2**  **Correlation between the intensity of ApoE expression and the clinical profiles in stage II CRC cohort.** | | | | | |
| --- | --- | --- | --- | --- | --- |
| **Factor** | Total | APOE-HIGH (%) | APOE-LOW (%) | Statistics | p value |
| **Age(years)** |  |  |  |  |  |
| ＜65 | 216 | 78(36.1) | 138(63.9) | 0.845 | 0.358 |
| ≥65 | 90 | 27(75.6) | 63(24.4) |  |  |
| **Gender** |  |  |  |  |  |
| Male | 183 | 57(31.1) | 126(68.9) | 2.025 | 0.155 |
| Female | 123 | 48(39.0) | 75(61.0) |  |  |
| **Tumor location** |  |  |  |  |  |
| Colon | 143 | 55(38.5) | 88(61.5) | 2.049 | 0.152 |
| Rectum | 163 | 50(30.7) | 113(69.3) |  |  |
| **Gross Pathological Type** |  |  |  |  |  |
| Prominence | 171 | 58(33.9) | 113(66.1) | 0.027 | 0.870 |
| Ulceration& Infiltration | 135 | 47(34.8) | 88(65.2) |  |  |
| **Grade** |  |  |  |  |  |
| High | 34 | 11(32.4) | 23(67.6) | 0.066 | 0.968 |
| Middle | 240 | 83(34.6) | 157(65.4) |  |  |
| Low | 32 | 11(34.4) | 21(65.6) |  |  |
| **T stage** |  |  |  |  |  |
| T3 | 286 | 98(34.3) | 188(65.7) | 0.004 | 0.947 |
| T4 | 20 | 7(35.0) | 13(65.0) |  |  |
| **Neurological involvement** |  |  |  |  |  |
| Present | 18 | 8(44.4) | 10(55.6) | 0.871 | 0.351 |
| Absent | 288 | 97(33.7) | 191(66.3) |  |  |
| **Vascular invasion** |  |  |  |  |  |
| Present | 11 | 5(45.5) | 6(54.5) | 0.220^*^ | 0.639^*^ |
| Absent | 295 | 100(33.9) | 195(66.1) |  |  |
| **Adjuvant therapy** |  |  |  |  |  |
| Chemotherapy | 131 | 45(34.4) | 86(65.6) | 0.744 | 0.689 |
| Radiotherapy | 63 | 19(30.2) | 44(69.8) |  |  |
| No | 112 | 41(36.6) | 71(63.4) |  |  |
| **CEA level（ng/ml）** |  |  |  |  |  |
| ≤5 | 244 | 84(34.4) | 160(65.6) | 0.007 | 0.934 |
| ＞5 | 62 | 21(33.9) | 41(66.1) |  |  |
| **CA19-9 level（U/ml）** |  |  |  |  |  |
| ≤37 | 282 | 98(34.6) | 185(65.4) | 0.166 | 0.684 |
| ＞37 | 23 | 18(30.4) | 5(69.6) |  |  |
| **MSI** |  |  |  |  |  |
| MSS | 268 | 90(76.5) | 178(23.5) | 0.513 | 0.474 |
| MSI | 38 | 15(39.5) | 23(60.5) |  |  |
| * represents adjusted Chi-square statistic result. | | | | | |
