# Supplementary material for: Apolipoprotein E Overexpression Is Associated With Tumor Progression and Poor Survival in Colorectal Cancer
Source: Front Genet. 2018 Dec 13;9:650. doi: 10.3389/fgene.2018.00650 (PMC6315167; doi:10.3389/fgene.2018.00650)
Supplement: Supplementary file 3 [file Table_3.DOCX]

| **Table S3**  **Correlation between the intensity of ApoE expression and the clinical profiles of simultaneous liver metastatic colorectal cancer cohort.** | | | | | |
| --- | --- | --- | --- | --- | --- |
| **Factor** | Total | APOE-HIGH (%) | APOE-LOW (%) | Statistics | p value |
| **Age(years)** |  |  |  |  |  |
| ＜65 | 153 | 79(51.6) | 74(48.4) | 0.039 | 0.843 |
| ≥65 | 48 | 24(50.0) | 24(50.0) |  |  |
| **Gender** |  |  |  |  |  |
| Male | 128 | 63(49.2) | 65(50.8) | 0.428 | 0.513 |
| Female | 73 | 40(54.8) | 33(45.2) |  |  |
| **Tumor location** |  |  |  |  |  |
| Colon | 109 | 57(52.3) | 52(47.7) | 0.105 | 0.746 |
| Rectum | 92 | 46(50.0) | 46(50.0) |  |  |
| **Gross Pathological Type** |  |  |  |  |  |
| Prominence | 78 | 38(48.7) | 40(51.3) | 0.325 | 0.568 |
| Ulceration& Infiltration | 123 | 65(52.8) | 58(47.2) |  |  |
| **Grade** |  |  |  |  |  |
| High& Middle | 157 | 82(52.2) | 75(47.8) | 0.015 | 0.901 |
| Low | 43 | 22(51.2) | 21(48.8) |  |  |
| **T stage** |  |  |  |  |  |
| T1&T2&T3 | 117 | 48(41.0) | 69(59.0) | 3.096 | 0.079 |
| T4 | 84 | 45(53.6) | 39(46.4) |  |  |
| **N stage** |  |  |  |  |  |
| N-positive | 159 | 82(51.6) | 77(48.4) | 0.033 | 0.856 |
| N-negative | 42 | 21(50.0) | 21(50.0) |  |  |
| **Neoadjuvant Therapy** |  |  |  |  |  |
| Yes | 59 | 38(64.4) | 21(35.6) | 11.052 | 0.001 |
| No | 142 | 55(38.7) | 87(61.3) |  |  |
| **Chemotherapy** |  |  |  |  |  |
| Yes | 165 | 80(48.5) | 85(51.5) | 1.820 | 0.177 |
| No | 36 | 13(36.1) | 23(63.9) |  |  |
| **preoperative CEA level（ng/ml）** |  |  |  |  |  |
| ≤5 | 66 | 30(45.5) | 36(54.5) | 0.026 | 0.871 |
| ＞5 | 135 | 63(46.7) | 72(53.3) |  |  |
| **preoperative CA19-9 level（U/ml）** |  |  |  |  |  |
| ≤37 | 127 | 63(49.6) | 64(50.4) | 1.546 | 0.214 |
| ＞37 | 74 | 30(40.5) | 44(59.5) |  |  |
| **MSI** |  |  |  |  |  |
| MSS | 185 | 98(53.0) | 87(47.0) | 2.781 | 0.095 |
| MSI | 16 | 5(31.3) | 11(68.8) |  |  |
