# Supplementary material for: Apolipoprotein E Overexpression Is Associated With Tumor Progression and Poor Survival in Colorectal Cancer
Source: Front Genet. 2018 Dec 13;9:650. doi: 10.3389/fgene.2018.00650 (PMC6315167; doi:10.3389/fgene.2018.00650)
Supplement: Supplementary file 4 [file Table_4.DOCX]

| **Table S4**  **Cox analyses of potential prognostic factors for overall survival in the stage II CRC cohort** | | | | | | | | |
| --- | --- | --- | --- | --- | --- | --- | --- | --- |
| **Factor** | Comparison | Univariate Analysis | | |  | Multivariate Analysis | | |
|  |  | HR | 95%CI | *p* value |  | HR | 95%CI | *p* value |
| **Age(years)** | ＜65 vs. ≥65 | 1.502 | 0.949-2.379 | 0.083 |  |  |  |  |
| **Gender** | Female vs. Male | 0.901 | 0.721-1.125 | 0.356 |  |  |  |  |
| **Tumor Location** | Colon Cancer vs.  Rectal Cancer | 0.940 | 0.752-1.174 | 0.585 |  |  |  |  |
| **Gross Pathological Type** | Prominence vs.  Ulceration & Infiltration | 1.312 | 0.837-2.057 | 0.237 |  |  |  |  |
| **T stage** | T3 vs. T4 | 1.493 | 0.687-3.247 | 0.312 |  |  |  |  |
| **Grade** | High & Middle vs. Low | 1.013 | 0.548-1.874 | 0.967 |  |  |  |  |
| **Neurological Involvement** | Present vs. Absent | 1.731 | 0.796-3.763 | 0.166 |  |  |  |  |
| **Vascular Invasion** | Present vs. Absent | 0.553 | 0.206-1.484 | 0.240 |  |  |  |  |
| **Adjuvant Therapy** | Yes vs. No | 0.821 | 0.528-1.278 | 0.383 |  |  |  |  |
| **Chemotherapy** | Yes vs. No | 1.045 | 0.648-1.688 | 0.856 |  |  |  |  |
| **CEA level（ng/ml）** | ≤5 vs.＞5 | 0.925 | 0.534-1.602 | 0.780 |  |  |  |  |
| **CA19-9 level（U/ml）** | ≤37 vs.＞37 | 0.675 | 0.247-1.847 | 0.444 |  |  |  |  |
| **MSI status** | MSI vs. MSS | 0.343 | 0.126-0.939 | 0.037 |  | 0.328 | 0.120-0.897 | 0.030 |
| **APOE expression** | HIGH vs. LOW | 1.973 | 1.266-3.077 | 0.003 |  | 2.023 | 1.297-3.154 | 0.002 |
