# Supplementary material for: Apolipoprotein E Overexpression Is Associated With Tumor Progression and Poor Survival in Colorectal Cancer
Source: Front Genet. 2018 Dec 13;9:650. doi: 10.3389/fgene.2018.00650 (PMC6315167; doi:10.3389/fgene.2018.00650)
Supplement: Supplementary file 5 [file Table_5.DOCX]

| **Table S5**  **Cox analyses of potential prognostic factors for progression-free survival in the stage II CRC cohort** | | | | | | | | | |
| --- | --- | --- | --- | --- | --- | --- | --- | --- | --- |
| **Factor** | Comparison | Univariate Analysis | | |  | | Multivariate Analysis | | |
|  |  | HR | 95%CI | *p* value |  | HR | | 95%CI | *p* value |
| **Age(years)** | ＜65 vs. ≥65 | 1.163 | 0.781-1.730 | 0.458 |  |  | |  |  |
| **Gender** | Female vs. Male | 0.926 | 0.635-1.351 | 0.690 |  |  | |  |  |
| **Tumor Location** | Colon Cancer vs. Rectal Cancer | 0.966 | 0.802-1.164 | 0.717 |  |  | |  |  |
| **Gross Pathological Type** | Prominence vs.  Ulceration & Infiltration | 1.194 | 0.820-1.738 | 0.356 |  |  | |  |  |
| **T Stage** | T3 vs. T4 | 1.337 | 0.676-2.644 | 0.404 |  |  | |  |  |
| **Grade** | High & Middle vs. Low | 1.078 | 0.643-1.806 | 0.777 |  |  | |  |  |
| **Neurological Involvement** | Present vs. Absent | 2.222 | 1.191-4.145 | 0.012 |  | 2.115 | | 1.133-3.949 | 0.019 |
| **Vascular Invasion** | Present vs. Absent | 0.948 | 0.575-1.562 | 0.834 |  |  | |  |  |
| **Adjuvant Therapy** | Yes vs. No | 0.908 | 0.626-1.318 | 0.613 |  |  | |  |  |
| **Chemotherapy** | Yes vs. No | 1.092 | 0.723-1.650 | 0.675 |  |  | |  |  |
| **CEA level（ng/ml）** | ≤5 vs.＞5 | 1.088 | 0.697-1.698 | 0.711 |  |  | |  |  |
| **CA19-9 level（U/ml）** | ≤37 vs.＞37 | 0.720 | 0.316-1.640 | 0.435 |  |  | |  |  |
| **MSI status** | MSI vs. MSS | 0.625 | 0.326-1.197 | 0.157 |  |  | |  |  |
| **APOE expression** | HIGH vs. LOW | 1.913 | 1.317-2.780 | 0.001 |  | 1.883 | | 1.295-2.737 | 0.001 |
