# Supplementary material for: Apolipoprotein E Overexpression Is Associated With Tumor Progression and Poor Survival in Colorectal Cancer
Source: Front Genet. 2018 Dec 13;9:650. doi: 10.3389/fgene.2018.00650 (PMC6315167; doi:10.3389/fgene.2018.00650)
Supplement: Supplementary file 6 [file Table_6.DOCX]

| **Table S6**  **Cox analyses of potential prognostic factors for overall survival in the simultaneous liver metastatic CRC cohort** | | | | | | | | |
| --- | --- | --- | --- | --- | --- | --- | --- | --- |
| **Factor** | Comparison | Univariate Analysis | | |  | Multivariate Analysis | | |
|  |  | HR | 95%CI | *p* value |  | HR | 95%CI | *p* value |
| **Age(years)** | ＜65 vs. ≥65 | 1.165 | 0.794-1.709 | 0.434 |  |  |  |  |
| **Gender** | Female vs. Male | 0.756 | 0.538-1.062 | 0.107 |  |  |  |  |
| **Tumor Location** | Colon Cancer vs. Rectal Cancer | 0.980 | 0.830-1.157 | 0.813 |  |  |  |  |
| **Gross Pathological Type** | Prominence vs.  Ulceration& Infiltration | 1.095 | 0.777-1.544 | 0.603 |  |  |  |  |
| **T Stage** | T1-3 vs. T4 | 0.696 | 0.499-0.970 | 0.032 |  | 0.786 | 0.556-1.109 | 0.170 |
| **N Stage** | N0 vs. N+ | 0.502 | 0.312-0.808 | 0.005 |  | 0.488 | 0.302-0.789 | 0.003 |
| **Grade** | High & Middle vs. Low | 0.751 | 0.508-1.110 | 0.151 |  |  |  |  |
| **Neoadjuvant Therapy** | Yes vs. No | 0.893 | 0.616-1.297 | 0.553 |  |  |  |  |
| **Chemotherapy** | Yes vs. No | 0.641 | 0.429-0.956 | 0.029 |  | 0.766 | 0.512-1.146 | 0.195 |
| **Targeted Therapy** | Yes vs. No | 1.010 | 0.664-1.537 | 0.963 |  |  |  |  |
| **CEA level（ng/ml）** | ≤5 vs.＞5 | 0.789 | 0.551-1.130 | 0.196 |  |  |  |  |
| **CA19-9 level（U/ml）** | ≤37 vs.＞37 | 0.716 | 0.511-1.003 | 0.052 |  |  |  |  |
| **MSI** | MSI vs. MSS | 0.597 | 0.292-1.219 | 0.157 |  |  |  |  |
| **APOE expression** | HIGH vs. LOW | 1.629 | 1.163-2.281 | 0.005 |  | 1.559 | 1.096-2.216 | 0.013 |
