# Supplementary material for: Apolipoprotein E Overexpression Is Associated With Tumor Progression and Poor Survival in Colorectal Cancer
Source: Front Genet. 2018 Dec 13;9:650. doi: 10.3389/fgene.2018.00650 (PMC6315167; doi:10.3389/fgene.2018.00650)
Supplement: Supplementary file 7 [file Table_7.DOCX]

| **Table S7**  **Cox analyses of potential prognostic factors for progression-free survival in the simultaneous liver metastatic CRC cohort** | | | | | | | | |
| --- | --- | --- | --- | --- | --- | --- | --- | --- |
| **Factor** | Comparison | Univariate Analysis | | |  | Multivariate Analysis | | |
|  |  | HR | 95%CI | *p* value |  | HR | 95%CI | *p* value |
| **Age(years)** | ＜65 vs. ≥65 | 1.059 | 0.744-1.507 | 0.750 |  |  |  |  |
| **Gender** | Female vs. Male | 0.758 | 0.554-1.036 | 0.082 |  |  |  |  |
| **Tumor Location** | Colon Cancer vs. Rectal Cancer | 0.965 | 0.828-1.123 | 0.643 |  |  |  |  |
| **Gross Pathological Type** | Prominence vs.  Ulceration& Infiltration | 1.059 | 0.774-1.451 | 0.719 |  |  |  |  |
| **T stage** | T1-3 vs. T4 | 0.800 | 0.590-1.087 | 0.153 |  |  |  |  |
| **N stage** | N0 vs. N+ | 0.483 | 0.317-0.737 | 0.001 |  | 0.462 | 0.302-0.706 | <0.001 |
| **Grade** | High & Middle vs. Low | 0.769 | 0.537-1.103 | 0.153 |  |  |  |  |
| **Neoadjuvant Therapy** | Yes vs. No | 1.004 | 0.721-1.004 | 0.979 |  |  |  |  |
| **Chemotherapy** | Yes vs. No | 0.776 | 0.530-1.136 | 0.192 |  |  |  |  |
| **Targeted Therapy** | Yes vs. No | 1.378 | 0.955-1.988 | 0.086 |  |  |  |  |
| **CEA level（ng/ml）** | ≤5 vs. ＞5 | 0.980 | 0.708-1.355 | 0.902 |  |  |  |  |
| **CA19-9 level（U/ml）** | ≤37 vs. ＞37 | 0.840 | 0.615-1.148 | 0.273 |  |  |  |  |
| **MSI** | MSI vs. MSS | 0.497 | 0.262-0.942 | 0.032 |  | 0.555 | 0.291-1.057 | 0.073 |
| **APOE expression** | HIGH vs. LOW | 1.496 | 1.100-2.033 | 0.010 |  | 1.541 | 1.129-2.104 | 0.006 |
